# Model-Based and Model-Free Replay Mechanisms for Reinforcement Learning in Neurorobotics

**DOI:** 10.3389/fnbot.2022.864380

**Published:** 2022-06-24

**Authors:** Elisa Massi, Jeanne Barthélemy, Juliane Mailly, Rémi Dromnelle, Julien Canitrot, Esther Poniatowski, Benoît Girard, Mehdi Khamassi

**Affiliations:** Institut des Systèmes Intelligents et de Robotique, UMR 7222, CNRS, Sorbonne Université, Paris, France

**Keywords:** hippocampal replay, reinforcement learning, neurorobotics, model-based, model-free

## Abstract

Experience replay is widely used in AI to bootstrap reinforcement learning (RL) by enabling an agent to remember and reuse past experiences. Classical techniques include shuffled-, reversed-ordered- and prioritized-memory buffers, which have different properties and advantages depending on the nature of the data and problem. Interestingly, recent computational neuroscience work has shown that these techniques are relevant to model hippocampal reactivations recorded during rodent navigation. Nevertheless, the brain mechanisms for orchestrating hippocampal replay are still unclear. In this paper, we present recent neurorobotics research aiming to endow a navigating robot with a neuro-inspired RL architecture (including different learning strategies, such as model-based (MB) and model-free (MF), and different replay techniques). We illustrate through a series of numerical simulations how the specificities of robotic experimentation (e.g., autonomous state decomposition by the robot, noisy perception, state transition uncertainty, non-stationarity) can shed new lights on which replay techniques turn out to be more efficient in different situations. Finally, we close the loop by raising new hypotheses for neuroscience from such robotic models of hippocampal replay.

## 1. Introduction

For a reinforcement learning (RL) agent (Lin, 1992; Sutton and Barto, 1998), experience replay consists of storing in (episodic) memory a buffer containing a series of observations (i.e., a quadruplet composed of the previous state, the action, the new state, and the reward) and periodically replaying elements from this buffer to bootstrap learning during offline phases (i.e., between phases where the agent acts and samples new observations in the real-world) (Fedus et al., 2020).

Several important parameters have an impact on the performance of RL agents with experience replay, such as the size of the memory buffer (Zhang and Sutton, 2017), the relative time spent learning from replay vs. the time spent collecting new observations in the world (Fedus et al., 2020), or whether to shuffle the memory buffer and uniformly sample elements from it or prioritize elements as a function of their associated level of surprise (e.g., the absolute reward prediction error associated to a given quadruplet observed from the environment) (Moore and Atkeson, 1993; Peng and Williams, 1993; Schaul et al., 2015).

To our knowledge, these replay techniques have their origin in the 1990s, when Long-Ji Lin at Carnegie Mellon University proposed solutions to enable RL reactive agents [i.e., model-free (MF) agents such as Q-learners Watkins, 1989] to bootstrap their learning process in large dynamic (non-stationary) discrete simulation environments (Lin, 1992). One of the investigated solutions was to use the Dyna-Q architecture (Sutton, 1990) to learn action models and use these models to sample hypothetical actions. Another tested solution consisted of storing the agent's experience in a memory buffer and replaying it to bootstrap learning. Interestingly, one of the main results was that the best performance was obtained by reversing the order of the replay buffer, what we will call *backward replay* (i.e., replaying first the most recent observation, then the second-to-last one, and so on until the oldest observation). This is because each time the buffer contains a rewarding observation, replay leads to increasing the value of the action performed in the previous state, followed by replaying precisely that previous state at the next iteration (because the buffer is in reverse order), and thus increasing the value of the preceding action, and so on. As a consequence, single processing of the memory buffer results in reward value propagation from rewarding states along the whole sequence of actions that the agents had experienced to get the reward.

In parallel, other researchers further investigated the efficiency of model-based (MB) techniques to sample hypothetical actions rather than replaying experienced actions from a memory buffer. One example is *prioritized sweeping* and consists of replacing uniform model sampling with a prioritization that depends on the absolute value of the reward prediction error (Moore and Atkeson, 1993; Peng and Williams, 1993). While MB methods can be conceived as ways of planning, thus different from MF learning, they can nevertheless be seen as an alternative way to perform offline Q-value updates. Even further, there is a mathematical equivalence between the sequence of Q-values obtained with MB updates and with MF methods with replay (van Seijen and Sutton, 2015). This is why throughout this paper, we will discuss both *model-based* and *model-free replay*, in the sense that they represent alternative offline reactivation mechanisms to update action values. We will refer to model sampling as *Simulation Reactivations* (SimR) and sampling from a memory buffer as *Memory Reactivations* (MemR).

Strikingly, neuroscience research has found that the mammalian brain also seems to perform some sort of experience-dependent reactivations of neural activity, in particular, in a part of the brain called the *hippocampus* (Wilson and McNaughton, 1994). These reactivations occur either when an animal is sleeping (Ji and Wilson, 2007) or during moments of quiet wakefulness between trials of the task (Karlsson and Frank, 2009). Most importantly, these reactivations play an instrumental role in learning and memory consolidation, since blocking these neural reactivations leads to impaired learning performance (Girardeau et al., 2009; Ego-Stengel and Wilson, 2010; Jadhav et al., 2012), while new memories can be created by stimulating reward circuits during these reactivations (De Lavilléon et al., 2015).

The computational neuroscience literature has recently compared the different replay techniques from machine learning with the properties of hippocampal replay recorded experimentally (Pezzulo et al., 2017; Cazé et al., 2018; Mattar and Daw, 2018; Khamassi and Girard, 2020). Interestingly, the reactivation of a sequence of states experienced by the animal during the task sometimes occurs in the same *forward* order and sometimes in *backward* order (Foster and Wilson, 2006; Diba and Buzsáki, 2007). Nevertheless, a large part of hippocampal reactivations occur in apparent random order, and the underlying computational principle remains to be explained (see for instance the proposal of Aubin et al., 2018). Moreover, computational investigations recently found that prioritized sweeping can also explain some properties of hippocampal reactivations (Cazé et al., 2018; Mattar and Daw, 2018). But it is not yet clear whether a single unified computational principle can explain hippocampal replay or whether the brain alternates between different types of replay (backward, shuffled, prioritized/MF vs. MB) in different situations (sleep vs. quiet wakefulness, depending on the difficulty of the task, the level of noise/uncertainty).

Thus, a new field of neurorobotics research is currently dedicated to integrating offline reactivations in the RL processes to improve and speed them up. As mentioned above, this focus on offline reactivations is both inspired by the machine learning techniques created in the 90s and now commonly used in DeepRL and by the neuroscience results on hippocampal reactivations and the probable cohabitation of MB and MF RL systems in the brain. With robotic applications as an aim, these contributions need to bridge the gap between perfectly controlled discrete state simulations and real embodied robotics experiments in continuous environments. The goal of this research is to understand which replay techniques give the best learning performance in different situations (constrained corridor-based vs. open maze environments; non-stationary goal locations and maze configurations) and whether robotic tests lead to different conclusions than simple perfectly controlled simulations (physical vs. abstract simulations, autonomous state decomposition by the robot, noisy perception). For instance, a recent neural network-based simulation of a rat maze task highlighted that shuffled experience replay was required to break the data temporal correlations to be able to learn a neural internal world model (Aubin et al., 2018). Importantly, while neurorobotics research during the last 20 years had already studied hippocampus models for robot navigation (Arleo and Gerstner, 2000; Fleischer et al., 2007; Dollé et al., 2008; Milford and Wyeth, 2010; Caluwaerts et al., 2012; Jauffret et al., 2015), to our knowledge, the impact of different types of replay on the performance of these models has only recently started to be investigated.

In this paper, we illustrate this line of research by presenting a series of numerical simulations of laboratory mazes (used to study rat navigation in neuroscience) as benchmark tasks for robotic learning. These simulations are presented in order of increasing complexity toward real-world robotic experiments. At each step of this presentation, we simulate and compare different replay techniques in either MF or MB RL agents. We discuss the properties of these simulations, how they contribute to improving learning in robots, and how they can also help generate predictions for neuroscience.

## 2. Simulation of Individual Replay Strategies in a Predefined Discrete State Space

**Figure 1:**
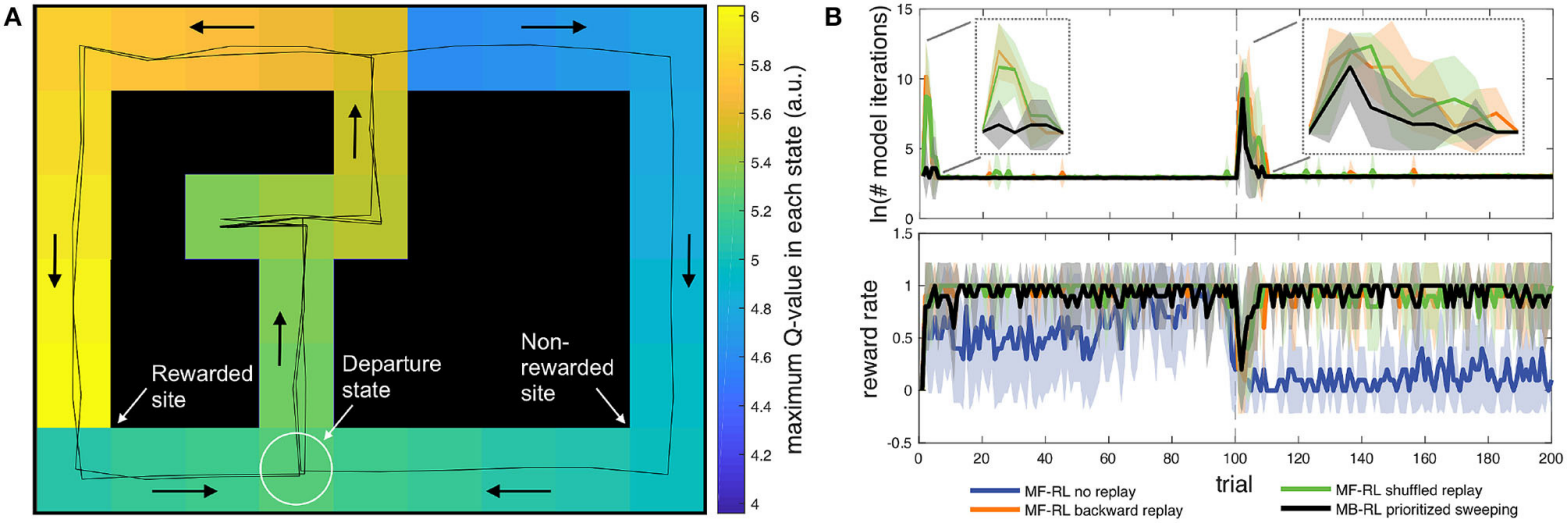
**(A)** Discrete state-space simulations in the multiple T maze task (Cazé et al., 2018; Khamassi and Girard, 2020). The reward is on the left side for 100 trials and then shifted to the right side for the next 100 trials. In the present simulations, replay is only allowed in the departure state, before starting the next trial. Despite this constraint, the figure shows that after only 3 trials (2 correct / 1 error), the MF-RL algorithm with backward replay has already learned a full gradient of Q-values across the maze. **(B)** Comparison of the performance (reward rate) and computation time (Napierian logarithm of the number of iterations during replay phases) for 4 different algorithms. The thick lines represent the average, and the area around represents the mean square error. The figure illustrates that MF-RL without replay requires 60–70 trials to reach optimal performance and does not manage to adapt to the change in reward location within only 100 trials. All the other algorithms perform similarly in terms of reward rate: fast increase in performance; brief drop in performance after the change in reward location; fast re-increase of performance afterward. These algorithms mainly differ in the required duration of the replay phases: MF-RL with random replay and MF-RL with backward replay both show a strong peak in the number of replay iterations after the change in goal location. The state-based version of MB-RL prioritized sweeping shows a smaller peak.

In this section, we present a series of numerical simulations in a simple deterministic maze task with predefined state decomposition. The task mimics the multiple T-maze of Gupta et al. (2010), where rats have to follow constrained corridors and make binary decisions (go left or go right) at specific T-like decision points (Figure 1A). This will enable us to first illustrate the properties of different replay methods in the same conditions as the perfectly controlled simulations usually performed in computational neuroscience work. Then in the next sections, we will study what happens in more open mazes where moreover the robot will autonomously build its state decomposition.

The work presented in this section contains two main differences from our previous computational neuroscience simulations of the multiple T-maze task (Cazé et al., 2018; Khamassi and Girard, 2020)^1^: (1) in previous work, following experience replay techniques in machine learning, we had allowed the agent to perform a series of replay iterations after each action; here, because it would be energy- and time-consuming for a robot to stop after each action, we allow the simulated robot to perform replay only at the end of the trial, while it is waiting for the next trial at the departure state; (2) we had simulated a version of *MB prioritized sweeping* where the memory buffer contained one element per state; here, we test whether it is also efficient to have an element for each (state,action) couple, thus filling the memory buffer with multiple elements for the same state (as long as they represent different actions).

### 2.1. Methods

We simulate the multiple T-maze task as a Markov decision problem (MDP), where an agent visits discrete states s∈S, using a finite set of discrete actions a∈A. States represent here unique locations in space, equally spaced on a square grid (Figure 1A), a piece of information expected to be provided by place cell activity in the hippocampus (O'Keefe and Dostrovsky, 1971). The actions allowed the agent to represent moves in the four cardinal directions: north, south, east, and west. During the first 100 trials, the reward will always be located on the left arm. Then during the next 100 trials, the reward will be on the right arm and the agent will have to adapt its decisions accordingly.

Here, we simulate three model-free RL algorithms and one MB one: MF without replay, MF with backward replay, MF with shuffled replay, and MB prioritized sweeping (Table 1).

**Table 1:** Algorithm parameters used to generate the results in this section.

	MF no replay	MF backward replay	MF shuffled replay	MB prioritized sweeping
α	0.2	0.2	0.2	-
γ	0.99	0.99	0.99	0.99
β	3	3	3	3
ϵ	-	0.001	0.001	0.001
*N*	-	54	54	54

*They have been taken from Cazé et al. (2018) without retuning. α is the model-free (MF) learning rate. γ is the discount factor. β is the inverse temperature in the softmax for decision-making (Equation 2). ϵ is the threshold for Q-values convergence during replay. N is the maximal size of the episodic memory buffer*.

For each Markovian state-action couple (*s, a*) in the environment, MF-RL agents use Q-learning (Watkins, 1989) to learn the Q-value of performing action *a* from state *s*, as follows:


(1) $$\begin{array}{r} Q\left(s,a\right)\leftarrow Q\left(s,a\right)+\alpha \left[R\left(s,a\right)+\gamma \underset{a^{\prime }}{\max}Q\left(s^{\prime },a^{\prime }\right)-Q\left(s,a\right)\right] \end{array}$$

Where *R*(*s, a*) is the reward obtained from the environment when performing (*s, a*), and *s*′ is the arrival state after executing action *a* in state *s*.

At the next timestep, deciding which action to perform is computed by drawing the next action *a* from a probability distribution given by the softmax Boltzmann function applied to the *Q* values:


(2) $$\begin{array}{r} P\left(a|s\right)=\frac{e^{\beta Q\left(s,a\right)}}{\sum_{i\in A}e^{\beta Q\left(s,i\right)}} \end{array}$$

With A being the set of all the possible actions from state *s* and β being the inverse temperature parameter that regulates the compromise between exploration and exploitation: the closer to zero, the more the differences between the Q-values will be attenuated, and thus the more the selection will be uniform (hence exploratory); conversely, large values (that can go up to infinity) will enhance the contrast between the Q-values and will thus favor exploitation of the largest one.

In *MF-RL backward replay* and *MF-RL shuffled replay* and for all the other RL replay algorithms tested in this section and the next one (Section 3), we enable the agent at each timestep to store in a memory buffer the quadruplet describing the current observation: the previous state *s* from which the agent performed action *a*, the resulting state *s*′ and the scalar reward *r* obtained from the environment (1 when the rewarding state has been reached, 0 elsewhere). This memory buffer progressively increases in size, timestep after timestep, but is limited by the maximal size *N* (*N* being chosen to correspond to the number of states in the environment, see Table 1). When the maximal size has been reached, adding a new element to the buffer is accompanied by throwing away the oldest element in it.

When the agent has finished the current trial and reaches the departure state again, a replay phase is initiated where at each replay iteration one element from the buffer is processed and the corresponding Q-value is updated following Equation 1. This is repeated until the sum of variations of Q-values over a window of *N* replay iterations is below a certain replay threshold ϵ, which indicates that the Q-values have converged and do not require to be updated anymore.

In the *MF-RL backward replay* algorithm (Lin, 1992), at the beginning of a new replay phase, we simply reverse the order of elements in the buffer and then start to perform replay iterations following the procedure explained above. In the *MF-RL shuffled replay* algorithm, we simply shuffle the elements of the buffer before starting the replay phase.

We also test an MB algorithm where the learning process aims at building a world model, i.e., a model of how the perceived world changes when actions are taken. This model is conventionally composed of a transition function and a reward function. The transition function *T*(*s, a, s*′) represents the probability of observing *s*′ next if action *a* is taken while in state *s*. In the present discrete case, it is built by storing the number of times each (*s, a, s*′) triplet was encountered and by dividing by the number of times (*s, a*) experienced, as shown in the equation below:


(3) $$\begin{array}{r} T\left(s,a,s^{\prime }\right)=\frac{V_{N}\left(s,a,s^{\prime }\right)}{V_{N}\left(s,a\right)} \end{array}$$

where *V*_*N*_(*s, a*) stands for the number of visits of state *s* when action *a* is then chosen and $V_{N}\left(s,a,s^{\prime }\right)$ is the number of transitions from state *s* to state *s*′, having performed action *a*. The reward function *R*(*s, a, s*′) represents the average reward signal experienced when effectively performing the (*s, a, s*′) transition. For the *MB-RL prioritized sweeping* algorithm that we simulate here (Moore and Atkeson, 1993; Peng and Williams, 1993), we add to each element in the memory buffer the absolute reward prediction error Δ measured when experiencing (*s, a, s*′, *r*) in world. This Δ can also be seen as representing the magnitude of change in *Q*(*s, a*) which resulted from this observation. The memory buffer is sorted in decreasing order of Δ, thus giving a high priority to be replayed to elements representing surprising events in the world that resulted in important revisions of Q-values. In fact, Mattar and Daw (2018) have formally shown that deriving the *Expected Value of (Bellman) Backup* (in other words an expected value of doing a replay) leads to maximizing a *gain* term which is higher for transitions that have been associated with larger reward prediction errors (hence larger surprise) when the agent was experiencing the real world.

During the replay phase of *MB-RL prioritized sweeping*, we start by considering the first element (*s, a*) of the buffer with the highest Δ. We use the world model learned by the agent to estimate the virtual reward *r* and arrival state *s*′, and then apply one iteration of the *Value Iteration* algorithm (Sutton and Barto, 1998) to update the Q-value of (*s, a*), where *k* is all the possible actions starting from the arriving state *s*′:


(4) $$\begin{array}{r} Q\left(s,a\right)\leftarrow R\left(s,a\right)+\gamma \underset{s^{\prime }}{\sum }T\left(s,a,s^{\prime }\right)max_{k\in A}Q\left(s^{\prime },k\right) \end{array}$$

From Equation 4, we can compute the new Δ for the couple (*s, a*) and reinsert it within the memory buffer with Δ as the new priority level. Finally, we use the world-model to find all possible predecessors of (*s, a*), i.e., couples (*s*″, *a*″), which according to the model enable the agent to reach state *s*. Because the predecessors of a given state *s* can be difficult to determine in a stochastic world, Moore and Atkeson (1993) propose to consider as predecessors all the states *s*″ which have, at least once in the history of the agent in the current task, performed a one-step transition *s*″ → *s*. The priority associated to a predecessor *s*″ can thus be the corresponding absolute prediction error Δ_*pred*_ and determined in which position it will be inserted in the memory buffer, as introduced by Peng and Williams (1993). The replay phase then continues by processing the next element in the buffer with the highest priority level, and so on, until one of the stop conditions described above is met. For the sake of terminological clarification, what we call here a replay phase for an MB algorithm corresponds to an inference phase. This is because *MB-RL prioritized sweeping* does not replay memorized past experience, but rather generates SimR through model sampling combined with the value iteration algorithm described above. Thus, to transpose from MF to MB, the replay phase stop conditions described above, the size of the replay budget *N* (which could also be called an inference budget in the case of MB) represents here a maximum number of iterations that can be inserted in the prioritized memory buffer and replayed through the value iteration algorithm.

### 2.2. Results

With the two changes that we made here compared (Cazé et al., 2018; Khamassi and Girard, 2020) (i.e., (1) only allowing the simulated robot to do replay at the end of the trial when reaching the departure state and (2) storing distinct (state, action) couple in the memory buffer for *MB-RL prioritized sweeping* rather than a single element per state), we found consistent performance results and only a difference in terms of a reduced computational cost during replay phases, which we describe below.

Figure 1B shows that the three algorithms with replay (*i.e., MF-RL backward replay, MF-RL shuffled replay*, and *MB-RL prioritized sweeping*) quickly reached the optimal reward rate of 1 at the beginning of learning and then experienced only a brief drop in reward rate after the change in reward location at trial #100. In contrast, *MF-RL without replay* took longer to reach the optimal rate (approx. 60 trials) and then barely managed to re-increase its reward rate within 100 trials after the change in reward location. So, the first conclusion is that any replay technique is equally useful in enabling fast learning in such a simple maze task with predefined state decomposition.

The second interesting observation has to do with the transient and nearly discrete increases in replay time that are produced in responses to task changes (Figure 1B). All replay techniques enable the agent to avoid spending time performing replay during the majority of the task. They moreover show a sharp increase in replay time after a change in reward location. Importantly, this property was also true in our previous work where replay was not restricted to the end of the trial but rather allowed in any state of the task (Cazé et al., 2018). Thus, it is interesting to note that such a way to generate replay events is not only compatible with neurobiological data (Cazé et al., 2018; Mattar and Daw, 2018) but also shows properties that could be useful for autonomous robots: bursts of replay could be used by the robot as a way to automatically detect new task conditions (but here the robot does not need to explicitly label these events; it just needs to adapt and maximize reward). The rest of the time, the agent starts each new trial without pausing, as if not showing any hesitation, similar to what is classically observed in well-trained rats in similar tasks (Gupta et al., 2010).

In addition, it is interesting to compare the duration of replay phases between the different replay techniques. While there is no difference in the average number of replay iterations after the change in reward location at trial #100 (Figure 1B), *MB-RL prioritized sweeping* performs drastically fewer replay iterations than *MF-RL backward replay* and *MF-RL shuffled replay* during the initial learning phase (first 5-10 trials of the task). Now that we restricted these algorithms to perform replay only at the end of each trial, rather than after each action during the trial, *MB-RL prioritized sweeping* performs even fewer replay iterations than what we previously obtained in the same task (Cazé et al., 2018), without affecting its reward rate. The new proposal to restrict replay to the inter-trial interval thus seems promising for real robots. In Dromnelle et al. (2020b) (where we had not implemented any replay mechanism yet), the robot indeed took a few seconds after each trial to go back to the departure state. This short moment seems ideal to let the algorithm perform a replay without affecting the performance of the robot during the trial.

In the next section, we keep these principles and compare the same replay algorithms in a more open environment where the robot autonomously learns to decompose the task into discrete states, to verify that these algorithms still perform well under these more realistic conditions.

## 3. Simulation of Individual Replay Strategies With an Autonomously Learned State Decomposition

The neural activity of hippocampal place cells is often observed as showing transients and increases after surprising events (Valenti et al., 2018). During maze navigation, surprising events mostly occur at locations in the environment that are associated with positive or negative outcomes. From these locations, reverse replay, in particular, could reinforce spatial learning by occurring during awake periods, after the spatial experiences (Foster and Wilson, 2006). They can potentially reinforce the surprising experience by propagating the outcome of the event to states that have been encountered by the animal on its way to the reward or punishment site. Moreover, rewarding states are also very likely to initiate replay activity in the hippocampus to enhance the memory consolidation of novel information (Michon et al., 2019). During these events, the reactivation of the hippocampus neural activity is thought to be initiated by rewarding outcomes to bind this positive unexpected experience to the events that preceded it (Singer and Frank, 2009).

To study these and others phenomena related to spatial navigation learning in rodents, one of the first and most relevant experimental protocols is the *Morris Water Maze* (Morris, 1981). In this work, rats were introduced to a circular pool with opaque water and were removed from the pool only after reaching a hidden platform, located just below the water surface. Even though the rats could not see the platform, they were still able to spatially localize it. This was found even in cases where their starting point changed within the pool, thus indicating a robust spatial memory.

In this section, the same MF-RL and MB-RL replay strategies (MemR and SimR, Sections 1, 2) are tested in a more realistic robotic set-up, where the discretization of the environment in multiple Markovian states is autonomously performed by the robot.^2^ Similarly to the experiment in Section 2 and to what has been experimentally observed by Foster and Wilson (2006), the replay phase takes place once the agent has reached the reward state to enable offline learning of Q-values, as previously done by Mattar and Daw (2018). Neurobiologically, even though *Vicarious Trial and Error (VTE*) plays an important role in animals' reasoning and decision-making (Tolman, 1939; Redish, 2016), it usually happens in uncertain moments, such as at beginning of the experiment, at the decision points or surprising spots (Cazé et al., 2018; Khamassi and Girard, 2020) and can also be unconsciously constrained by the attempt to limit the opportunity cost (Keramati et al., 2011).

This aspect is particularly crucial for the robotic experiment because it allows the agent to spend the Inter Trial Interval (ITI) updating the Q-table, based on a replay of its past experience. Usually, this time interval does not require expensive computations for the robot, since it does not need to take any decision on its way back to the starting position, and by replaying past experience, the learning speed could be enhanced without losing important experimental time.

The addressed research question is whether MF-RL or MB-RL replay strategies could enhance spatial learning for artificial agents and robots. We found it interesting to first test our proposed algorithm in a simulated version of an experimental task (Morris, 1981) and eventually investigate if there were any differences between replaying reverse sequences of actions, random transitions, or the most surprising transitions, similarly to what has been done in Section 2.

Like in the previous section, the presented simulated experiment investigates the role of diverse replay strategies relative to a changing reward condition. Moreover, the aim is also to investigate whether replays are relevant when transitions between the states of the task are stochastic. These simulations thus bring us to more realistic robotic experiments, in stochastic and dynamical environments.

### 3.1. Materials and Methods

To study the implications of offline learning in spatial navigation, from rodents' behavior to robotics, we have first investigated the role of two MF- and one MB-RL replay techniques (as in Section 2) in a circular maze, consistent with the original Morris water maze task (Morris, 1981) in terms of environment/robot size ratio. The learning performances of the analyzed replay techniques are discussed in two main conditions:

- A deterministic version of the task, where an action *a* performed in a state *s* will always lead the robot to the same arrival state *s*′ with probability 1.
- A stochastic version of the task, where performing action *a* in state *s* is associated with non-null probabilities of arriving in more than one state.

#### 3.1.1. Learning Algorithm and Replay

As in the previous series of simulations (Section 2), the simulated agent is learning using either classical *MF-RL Q-learning* (Watkins, 1989) (Equation 1) or *MB-RL prioritized sweeping learning* (Moore and Atkeson, 1993; Peng and Williams, 1993). The values of their parameters (learning rate α and the discount factor γ) are shown in Table 2.

**Table 2:** Algorithm parameters are used to generate the results in this section.

	No replay	MF backward replay	MF shuffled replay	MB prioritized sweeping
α	0.8	0.8	0.8	0.8
γ	0.9	0.9	0.9	0.9
β	15	15	15	15
ϵ	-	0.001	0.001	0.001
*N*	-	90	90	90

*α is the learning rate, optimized as shown in Figure 4 and Equation 6, and γ is the discount factor. β is the inverse temperature in the softmax function for decision-making (Equation 2), and its values were found by optimizing both the convergence time and the performance of the tested algorithms. N is the maximal length of the episodic memory buffer. This value was selected to replay the entire real experience during the first trials of the experiment and to replay experiences from several past trials later in the simulation. Finally, ϵ is the convergence threshold as for Sections 2 and Cazé et al. (2018)*.

The first implementation of offline learning techniques that we tested is the *MF backward replay*. Similar to the double T-maze experiment in Section 2, the offline learning phase happens once the agent has reached the reward state, which indicates the end of a trial. During a trial, the Q-values *Q*(*s, a*) of the state-action couple (*s, a*) are updated with Equation 1 and once the rewarding state has been reached, they are updated again in reverse order, starting from the reward state. These backward sequences can be up to *N* updates long if the agent has gained enough past experience and stored it in its memory buffer. The reverse sequences are then replayed until the sum of variations of Q-values over the last replay repetition is below a certain replay threshold ϵ (Table 2). Given the size of the environment (36 states), these *N* long backward replay sequences can also involve experiences that happened during the previous trials of the same agent (i.e., during the previous attempts to get to the reward). In this way, the robot can transfer the acquired knowledge through different trials and learn more efficiently.

The second replay strategy that has been tested is *MF shuffled replay*; in this case, in the ITI, the internal values *Q*(*s, a*) are randomly ordered and then updated by Equation 1. As for the *MF backward replay*, the memory buffer, that is accessible to initiate the reactivations, keeps in memory the latest *N* transitions (Table 2). Also, in this case, the agent can benefit from the experience acquired during the latest trials and learn to extract more general and useful knowledge from its recent and uncorrelated past actions (because of shuffling). The ITI replay phase lasts until the convergence of the sum of the Q-values under an ϵ value given in Table 2.

As for Section 2, we compared the learning performance of the above-explained MF replay strategies to an MB prioritized sweeping algorithm (Moore and Atkeson, 1993; Peng and Williams, 1993). The implementation of the latter is the same as described in Section 2.1, and the convergence criterion is reached when the prioritized replay buffer, which can be maximum *N* transitions long, is empty.

#### 3.1.2. The Experimental Set-Up and Implementation

**Figure 2:**
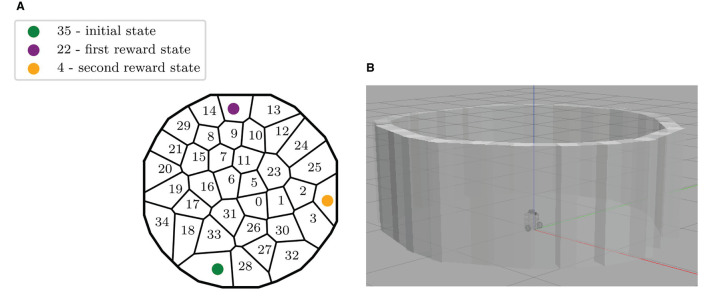
Description of the experimental set-up. **(A)** Map of the discrete states of the maze, identified by the robot during the exploration of Gazebo. The initial state and the two rewarding states are also highlighted. **(B)** The ROS Gazebo simulated Turtlebot 3 in the center of the circular environment.

The simulated experimental set-up intends to replicate a Morris water maze task: the agent is introduced in a new circular environment, and it has to learn how to reach a particular location associated with a positive reward (Morris, 1981). In our set-up, the agent is a Turtlebot3 Burger, simulated with the Robot Operating System (ROS) middleware and the Gazebo simulation environment (Quigley et al., 2009). The water maze is represented as an empty circular arena surrounded by high walls (Figure 2B).

The robot discovers and defines the different discretized areas in the maze by autonomously navigating within the environment. Despite the odometry and the laser sensor being installed on the robotic device, the acquired space representation is allocentric. This is an emergent property of the automatic clustering process when applied to robot sensor data in a task where the robot can only move in a horizontal plane, as found in previous neurorobotics work (Caluwaerts et al., 2012). The robot, in fact, explores by selecting between 8 directions of motion that are defined in the global reference frame of the environment, and its current position and orientation are also elaborated in the maze reference frame. This allocentric description of the robot movements and the states of the maze is possible thanks to a re-mapping of the relative position of the robotic agent and the discretized states to the reference coordinate system of the map. This is possible thanks to the 360 Laser Distance Sensor of the robotic platform, combined with the use of a classical SLAM technique. Note that such an allocentric space representation is not only compatible with neurophysiology (hippocampal place cell activity) but can also be combined with egocentric representations to account for a variety of experimentally observed animal behaviors during navigation tasks (Khamassi and Humphries, 2012). The discrete MDP, presented in Figure 2A, is obtained thanks to a Rao-Blackwellized particle filter that builds grid maps from laser range data (Grisetti et al., 2007). The simulated implementation of this Simultaneous Location and Mapping Algorithm (SLAM) on ROS Gazebo is called *GMapping*.

This state decomposition process makes the robot able to immediately create new states if necessary, but in our work, the aim was to create the finest and most robust possible discretization of the maze to be then employed in all the simulation experiments where we tested the different replay strategies. As observed by Khamassi (2007), Chaudhuri et al. (2019), and Benchenane et al. (2010), rats could re-explore the whole maze every day before doing a learning task and that could reflect their need to rapidly acquire and stabilize a state representation before starting an extra learning process.

For these reasons, the robot performs a long autonomous exploration phase to acquire its state representation before starting the learning phase. During the first 48-min-long exploration in Gazebo, the SLAM algorithm estimates the current robot coordinates, and whenever it is more than 15 cm further away from any existing state, it creates a new state, whose reference position is the current position. This results in a Voronoi partition of the space, composed here of 36 states (Figure 2A). This 15 cm state radius was chosen to be similar to the robot footprint of 13,8 x 17,8 x 19,2 (L x W x H, cm). The action space A instead contains 8 homogeneously distributed directions of motion, defined with respect to the world reference frame (same as for Section 4, **Figure 8A**, top right).

Then, we ran another free exploration of the arena by the simulated Turtlebot3 robot to automatically learn the transition probabilities *p*(*s*′|*s, a*) that can be approximated from randomly executing different actions *a* in different states *s* and observing the arrival state *s*′. This second free exploration phase was chosen to be 5,357-action long, the same duration as for the results that will be presented in Section 4. Lesaint et al. (2014) found that when an agent was progressively learning its transition function during the task, the RL model was better at accounting for rat behavior than a model with a prior given transition function.

In practice, the transition probabilities autonomously learned by the robot during free exploration in Gazebo is stochastic: the same action *a* performed in the same state *s* can lead to more than one state with non-null probabilities. For instance, moving north from state #31 alternatively leads to states #0,5,6,16, and even sometimes to state #31 itself when the robot initiated its movement from the bottom part of this state (Figure 2A). Such stochasticity results from several properties: (1) because the states autonomously decomposed by the algorithm are not evenly distributed; (2) because the experiments are performed in a simulated physical environment, which includes frictions between the robot's wheels and the floor, and where the robot sometimes moves too close to the walls, thus triggering its obstacle-avoidance process, hence resulting in a different effect of the same action performed without obstacle-avoidance.

The actual level of uncertainty of the stochastic version of the task is displayed in **Figure 10A**, where each state *s* has an entropy *H*_*env*_(*s*) computed as in Equation 5, where A is the set of all the possible actions *a* from state *s*, *s*′ are all the possible arrival states from the original state *s*, and *p*(*s*′|*s, a*) is the probability that the agent arrives in state *s*′ after starting from state *s* and performing action *a*:


(5) $$\begin{array}{r} H_{env}\left(s\right)=\underset{a\in A}{\max}\underset{s^{\prime }}{\sum }-p\left(s^{\prime }|s,a\right)\log_{2}p\left(s^{\prime }|s,a\right) \end{array}$$

**Figure 3:**
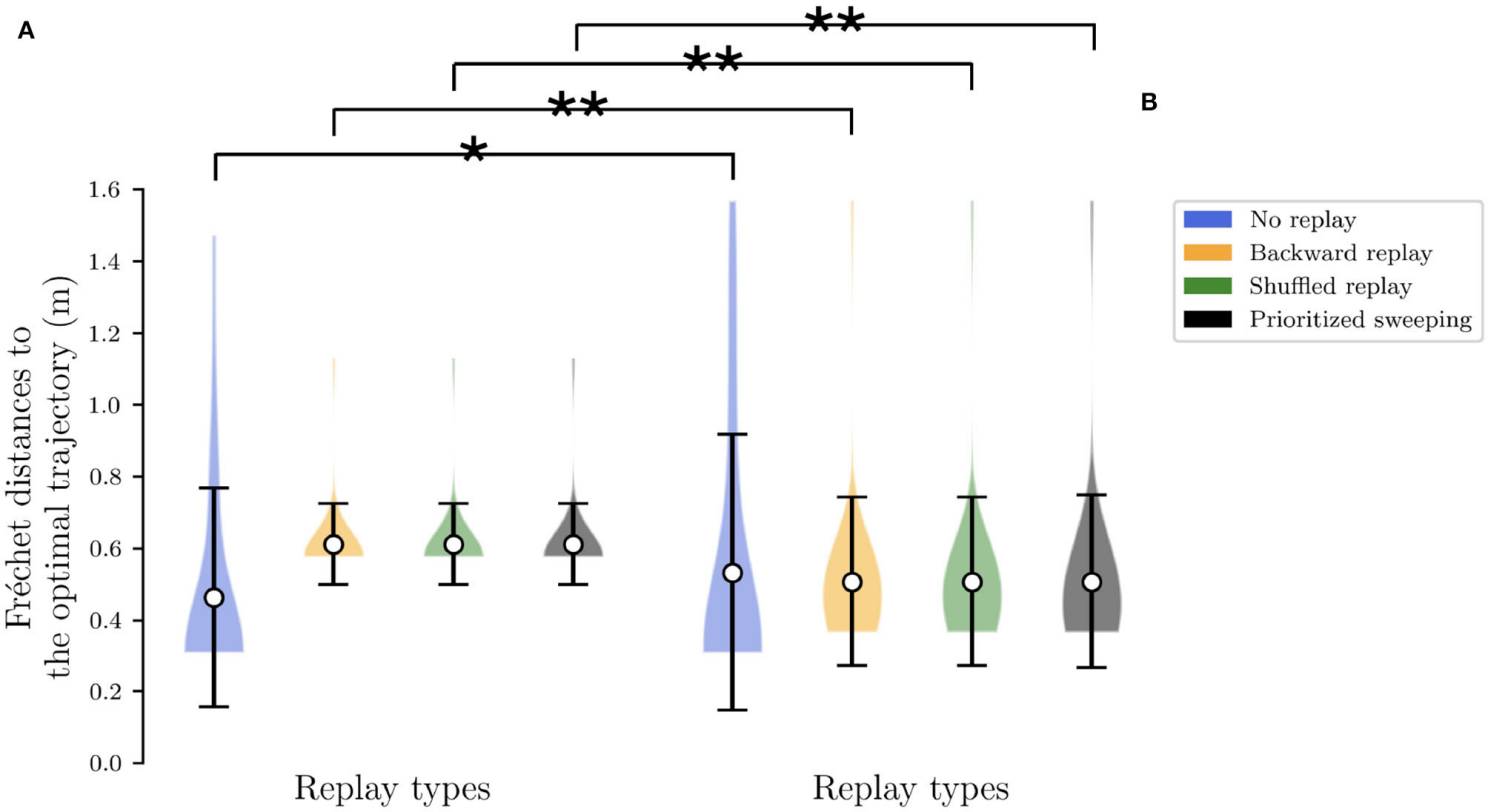
Analysis to investigate the level of the sparsity of the explored trajectories by the agent. The Fréchet distance has been computed for the first half of the simulation. ** Stands for *p*-value lower than 0.001 and * for *p*-value lower than 0.05. **(A)** The extension of the Fréchet distance to the optimal trajectory in the deterministic case for all the algorithms. **(B)** The same extension of Fréchet distance in the stochastic environment.

Finally, in order to obtain a deterministic version of the same task from these autonomously learned transition probabilities *p*(*s*′|*s, a*), for each (state, action) couple (*s, a*), we search for the state *s*′ with the highest probability of arrival (i.e., $s^{\prime }=\underset{x\in S}{\mathrm{argmax}}\left[p\left(x|s,a\right)\right]$), and set *p*(*s*′|*s, a*) = 1 while setting *p*(*s*″|*s, a*) = 0 for all other states *s*″(*s*″ ≠ *s*′). The deterministic version of the task consists in fact the simplification of the interaction between the robot and the environment, meaning that the trajectories that the robot can cover in the same environment are reduced. To quantify the simplification of the resulting MDP, we have performed an analysis of the trajectories which have been taken by the four different algorithms, in the two different environments. We compute the pairwise Fréchet distance of these trajectories to the optimal one, found by following a greedy optimal policy. Figure 3 shows this analysis during the first half of the experiment when the reward is fixed in state #22. The results from this analysis show that, for all the adopted strategies, in the stochastic environment (Figure 3B), the sparsity of the trajectories around the optimal path is generally higher compared to the same deterministic case Figure 3A). To assess the difference among these distance distributions, we did a Kruskal-Wallis *H*-test (Kruskal and Wallis, 1952)), finding them significantly different from each of their corresponding distribution of in the other environment. The conversion of the environment in a deterministic MDP is then intrinsically limiting the level of exploration of the agents, resulting in two very different scenarios. However, it is very important to investigate this transition, given our intent to study the role of RL replay strategies in robotic navigation, from a theoretical to a more realistic robotic outline.

To replicate a non-stationary task similar to the one in the original experiment (Morris, 1981), we changed the reward location from state #22 to state #4 at trial 25, and we tested the learning performances of the agent with four different replay strategies (no replay, MF backward replay, MF shuffled replay, and MB prioritized sweeping) and in two different environments: a deterministic and a stochastic version of the task.

### 3.2. Results

**Figure 4:**
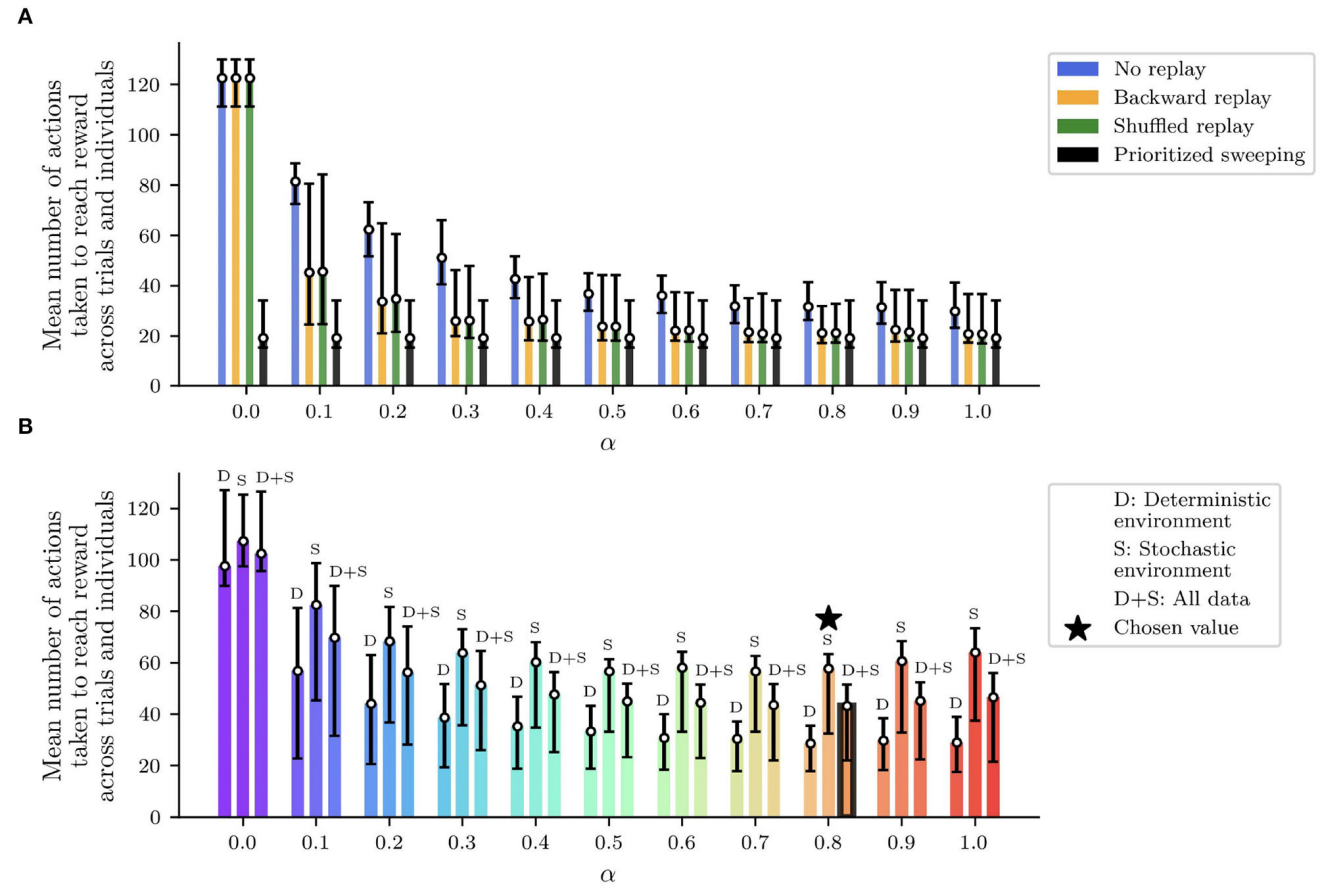
Performed analysis to find out the best learning rate α for all the replay strategies and the two environments (deterministic and stochastic). For different values of α, the figure shows the first, median, and third percentile of the number of actions to get to the reward, over 100 agents completing the simulated experiment over 50 trials. The average minimum number of model iterations to get to the reward is found for α equal to 0.8, and it was used for all the presented experiments (Table 2). **(A)** Performances of the tested algorithms across the α values in the deterministic version of the maze. **(B)** Final selection of α considering the mean performances between the deterministic and the stochastic version of the maze.

To assess the real contribution of the tested replay strategies to the learning process of the described spatial navigation task, an unbiased learning rate α_*best*_ has to be found. Since α_*best*_ could be different depending on the unpredictability of the MDP which simulates the task (i.e., deterministic or stochastic), we simulated 100 robotic agents performing 50 trials to get to the rewarding states, for a set of uniformly distributed α values between 0 and 1 (Figure 4). For each value of α, we looked at the average value *action*(α) along the trials, with *action*(α) being the number of actions needed by the robot to get to the rewarding states. These values are computed for both the deterministic (Figure 4A), the stochastic worlds, considering the entirety of the experiment, and the minimization of the sum of these two values is used to identify the final α_*best*_ (Figure 4B and Table 2) as described in the equation below:


(6) $$\begin{array}{r} \alpha_{best}=\underset{\alpha \in A}{\mathrm{argmin}}\left(\bar{action_{deterministic}\left(\alpha \right)}+\bar{action_{stochastic}\left(\alpha \right)}\right) \end{array}$$

where A is the set of tested α values.

Once identified the most appropriate value for the learning rate α, the following four replay conditions have been tested in the task:

- *MF-RL no replay*
- *MF-RL backward replay*
- *MF-RL shuffled replay*
- *MB-RL prioritized sweeping*

and the other relevant parameters for the experiment are described in Table 2.

**Figure 5:**
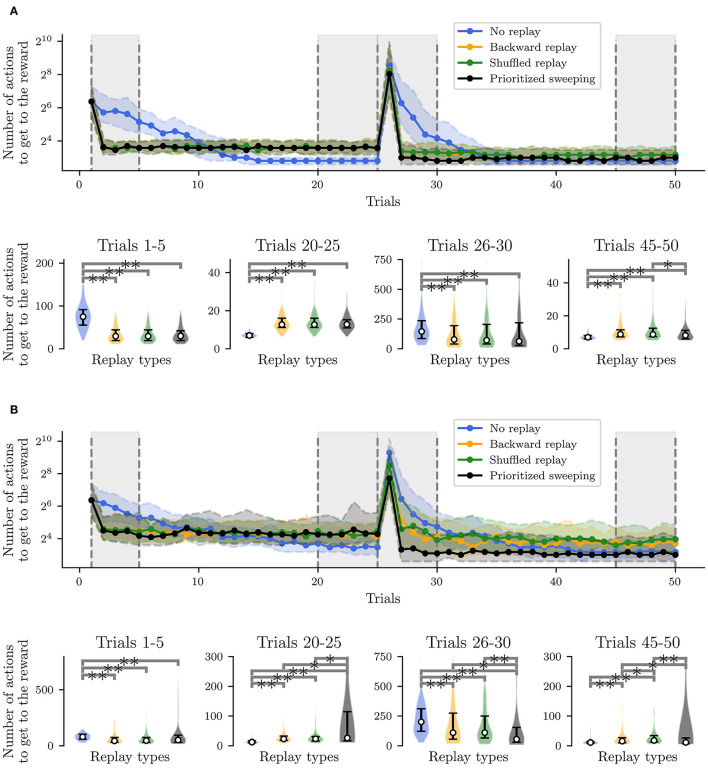
Performances of the simulated robot, learning the non-stationary task, and a *post-hoc* Wilcoxon-Mann-Whitney pairwise comparison test on the relevant trial intervals among the different curves. The *post-hoc* test has been performed following a Kruskal-Wallis *H*-test (Kruskal and Wallis, 1952) to reject the null hypothesis that the population median of all of the algorithms' average performances was equal. ** Stands for *p*-value lower than 0.001 and * for *p*-value lower than 0.05. **(A)** Deterministic environment. **(B)** Stochastic environment.

The main results are shown in Figure 5. The four different RL algorithms (no replay, backward replay, shuffled replay, and prioritized sweeping) are compared in terms of the number of model iterations to get to the rewarding state (Napierian logarithm of the first, median, and third percentiles over the behavior of 100 robotic agents). The task changes at trial #25 when the reward switches from state #22 to state #4 (Figure 2).

When the task is deterministic (Figure 5A), all three RL algorithms with replay learn a short path to the reward significantly faster than the *MF-RL no replay* learner (Figures 5A,B, Trials 1-5). The same situation occurs when the reward position is switched at trial #25, assessing the role of RL replays in improving the speed of learning after such a task change (Figures 5A,B, Trials 26-30). When the environment is stochastic, the situation is similar and, in particular, the prioritized sweeping algorithm is learning significantly faster than the other replay strategies (Figure 5B, Trials 26-30) reflecting the importance of an MB strategy (with MB replay) to faster adapt to dynamical tasks, when the transition model is not deterministic. This suggests that moving toward more complex robotic tasks, MB-RL models of replay may be preferred, since the higher information processing regarding the model of the environment, at the beginning of the task, can save real experimental time, when the robot would need to adapt later in the experiment.

Moreover, the logarithmic scale makes it easier to notice that the no replay agent, even if it is slower at the beginning of the task, can converge to paths that are significantly shorter than the one covered by the other strategies, before the change in reward location (Figures 5A,B, Trials 20-25). In the stochastic environment, in particular, the *MB-RL prioritized sweeping algorithm* reinforces the experience of a sub-optimal path, resulting in performance significantly different from the ones obtained from the other two replay strategies (Figure 5B, Trials 20–25). This shows that, even if the stochastic environment leads the MF-RL replay strategy to explore more the maze, the *MB-RL prioritized sweeping algorithm*, that can learn the transition model from the beginning of the task, is not subjected to this “push” toward exploration and keeps reinforcing the shortest path previously found.

Instead, in the second convergence phase (Trials 45–50), we highlight the fact that the no replay agent is not showing any more statistically better performances than all the replay algorithms (Figures 5A,B, Trials 45–50). In the deterministic case, it is still reaching the shortest path to the reward, but the prioritized sweeping agent is also being significantly better than the *MF-RL shuffled replay* strategy (Figure 5A Trials 45-50). On the other hand, in the stochastic case, the *MB-RL prioritized sweeping*'s knowledge of the environment makes it attain performances that are compatible with the ones from the no replay strategy. In this particular case, we can notice that the replay strategies perform differently, with the shuffled replay which performs worse than the other two replay strategies. This re-adaptation phase gives to the agents the opportunity for more exploration, in particular to the replay agents, which have strongly reinforced their previous experienced trajectory to maximize the reward and propagate this knowledge throughout the environment. As already happened in the second learning phase (Figure 5B, Trials 26–30), the *MB-RL prioritized sweeping algorithm* significantly exceeds the performance of the other replay algorithms and converges to a shorter path to the reward. This gives insights into the need for a more consolidated knowledge of the environment (and so of the interaction of the agent with it) for adaptive tasks. As a consequence, we can predict that animals would need to retrieve knowledge about their experienced and learned model of the world to adapt more efficiently to dynamic circumstances.

**Figure 6:**
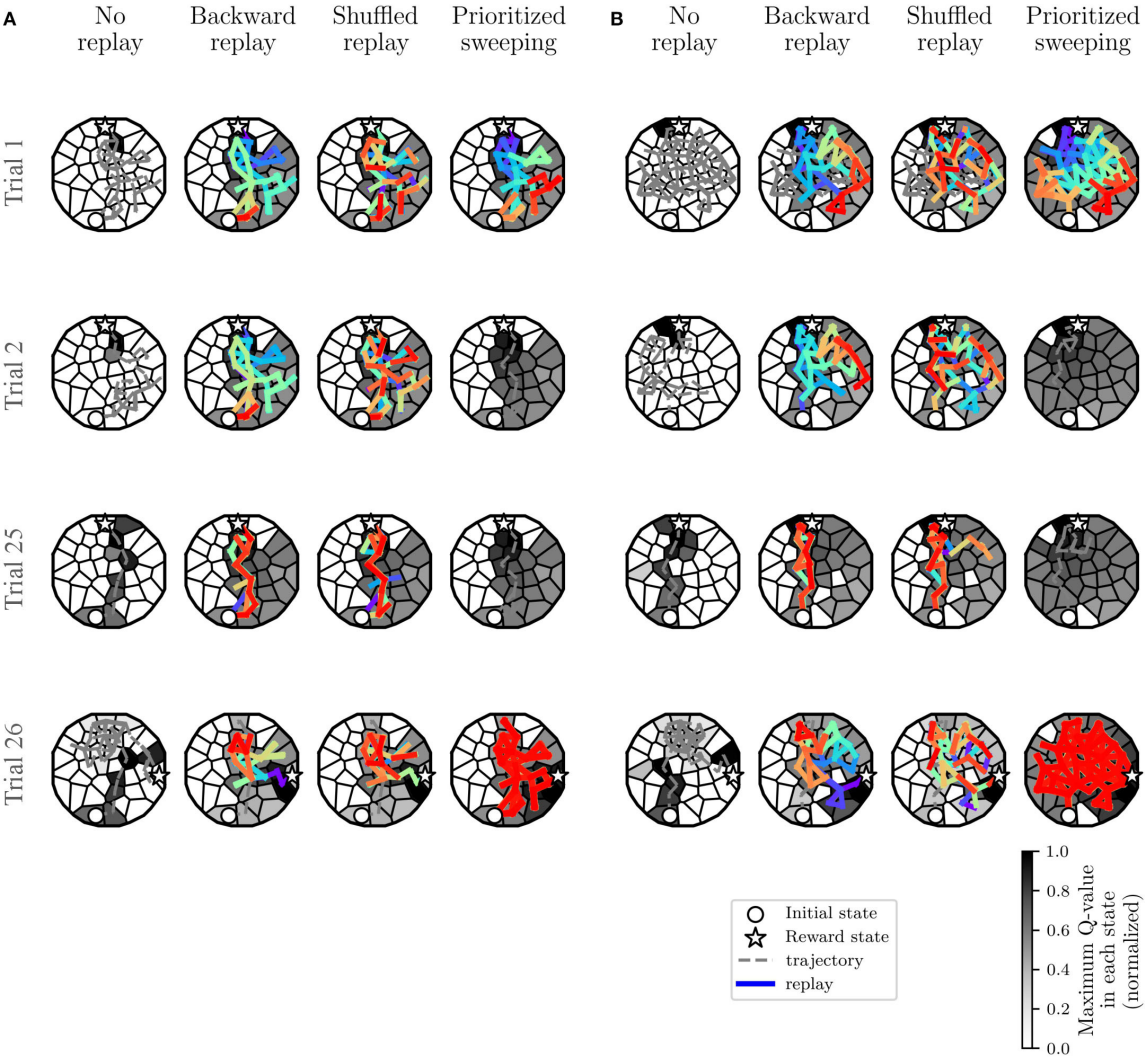
Learning dynamics of the most representative individual: covered trajectory and replay at some critical trials. Also, for each state *s*, the *maxQ*(*s, a*_*i*_), among all the *a*_*i*_, with *i* from 1 to 8 (Figure 8A, top right), is represented. The initial state and the reward state are also represented in the figure. **(A)** Experiments in the deterministic MDP. **(B)** Experiments in the stochastic MDP.

Following the results shown in Figure 5, we have further investigated the learning and replay dynamics of the proposed strategies. In Figure 6, the level of propagation of the Q-values (Equation 1) over the environment is shown for the different tested RL algorithms and for both the deterministic (Figure 6A) and the stochastic (Figure 6B) environments. The shown learning dynamics are representative of the different strategies since they show the behavior of the individual which is the closest to the median performances of all the 100 individuals for each strategy.

In both cases (Figures 6A,B, Trials 1,2 and 25), the presence of replay provides a drastically larger propagation of the Q-values, starting from the first reward state (22). This explains the significantly faster learning performances observed in the algorithms with replay compared to the *MF-RL no replay method*. In both environments, the no replay method is slower to learn, but it explores more in the first trials (Trial 1 and 2) and that leads it to generally find a shorter path to the reward location in the end (Trial 25) compared to the other learning strategies (as shown in Figure 5A and, Trials 20–25).

By comparing the two types of environments, we can understand that the level of stochasticity of the MDP leads to a more important exploration of the environment for all the strategies (Figure 6B, looking at the explored trajectories and the replayed transitions). This results in a larger propagation of the Q-values in the maze, in particular, in the prioritized sweeping algorithm. As in the deterministic case, the *MB-RL prioritized sweeping* is replaying a broader range of transitions after the first trial compared to the other strategies. With this MB-RL replay strategy, the replay activity is led by the surprise of the experienced events. This results in longer replay phases at specific surprising moments of the task, for instance after the first discovery of the reward location (at trial 1 in Figure 6) and after the discovery of the changed rewarding state (at trial 26 in Figure 6). This happens in both environments also thanks to the implementation of the algorithm which examine also the predecessor of the surprising state (Section 3.1.1) and to the acquired knowledge of the environment (in particular in Trials 26, when the reward position changes).

In both environments, as expected from the previously analyzed learning performance in Figure 5, there is no effective difference in terms of Q-value propagation between *MF-RL backward replay* and *MF-RL shuffled replay*. The explored trajectories and the replay are also very similar, resulting in not significantly different performances (Figure 5). These results, which simulate a spatial learning experiment for rodents (Morris, 1981) in a robotic framework, suggest some first advantageous properties of using replay-inspired strategies in neurorobotics. Our results imply that MF-RL replays could be sufficient to speed up learning and adaptation to non-stationarity (Figure 5, Trials 1–5 and 26–30), but MB-RL replay strategies could improve the adaptability of the system even more, with a higher level of stochasticity which often characterizes real robotic scenarios (Figure 5, Trials 26–30).

The proposed models and experiments contribute to a deeper understanding of the advantages and limitations of the existing RL models of replay in such robotics tasks. This experimental comparison, examining either a deterministic or stochastic version of the same environment (which implies a significantly different level of explored trajectories in the maze, see Figure 3) was useful to observe that RL replay gives an important contribution to a robotic spatial learning task, even if the model of the robot-environment interaction is stochastic. Nevertheless, a good compromise between the exploration capability of MF replay strategies and the adaptability of MB ones has not yet been found within these experiments. The next section will illustrate the performances of RL replay strategies in spatial learning when they are tested in combination in an MF-MB RL hybrid learning architecture in a more complex environment with obstacles, higher stochasticity, and non-stationarity.

## 4. Combining MB and MF Replay in a Changing Environment

Hippocampal replay has not only been interpreted as a memory consolidation process from past experience (Foster and Wilson, 2006; Girardeau et al., 2009), putatively MF, but also as a possible MB planning process that enables the mental simulation of hypothetical actions (Gupta et al., 2010; Ólafsdóttir et al., 2018; Khamassi and Girard, 2020). Along these lines, it has been argued that model sampling can not only be used for planning but also to update action values (van Seijen and Sutton, 2015; Cazé et al., 2018; Mattar and Daw, 2018). Moreover, some sequences of reactivated hippocampal neurons cannot be accounted for as a simple MF reactivation of past experience, and rather seem to represent creative combinations of past and experienced trajectories which can only be accounted for by a MB process (Gupta et al., 2010).

This suggests that both MF *MemR* and MB *SimR* are required to account for the diversity of hippocampal replays. Importantly, state-of-the-art models of RL processes in the mammalian brain assume a co-existence of MB and MF processes (Daw et al., 2005; Dollé et al., 2010, 2018; Keramati et al., 2011; Khamassi and Humphries, 2012; Pezzulo et al., 2013; Collins and Cockburn, 2020). Hence, neurorobotics constitutes a promising research area to study replay in robot control architectures that combine MB and MF RL processes.

With the experiments presented in the two previous sections, the complementary properties and performances of MF replay and MB replay have been analyzed. In our presented tasks, RL agents with MB replays tended to be slower to converge to an optimal solution but eventually, they reached a faster path to the reward location. On the other hand, the same agent with MF replay learned faster but converged to a suboptimal solution. In this section, in addition to pushing robot simulations toward more complex environments with stochasticity and non-stationarity, we want to examine the benefits of combining SimR and MemR in a robot control architecture which includes both MB and MF RL^3^. We thus investigate the effects of including replay in the algorithm proposed in Dromnelle et al. (2020b), which coordinates a MB and a MF RL expert within the decision layer of a robot control architecture. Interestingly, this algorithm had been previously tested in a navigation environment that includes open areas, corridors, dead-ends, a non-stationary task with changes in reward location, and a stochastic transition function between states of the task. In these conditions, previous results showed that the combination of MB and MF RL enables to (1) adapt faster to task changes thanks to the MB expert and (2) avoid the high computational cost of planning when the MF expert has been sufficiently trained by observation of MB decisions (Dromnelle et al., 2020b). Nevertheless, replay processes have not been included in this architecture yet, and the present paper is the opportunity to do it.

The results that we are going to illustrate and discuss in the following subsections present the combination of SimR and MemR as a critical resource to optimize the trade-off between the increase in performance and the reduction of computational cost in a hybrid MB-MF RL architecture when solving a more complex non-stationary navigation task than the two previous sections.

### 4.1. Materials and Methods

**Figure 7:**
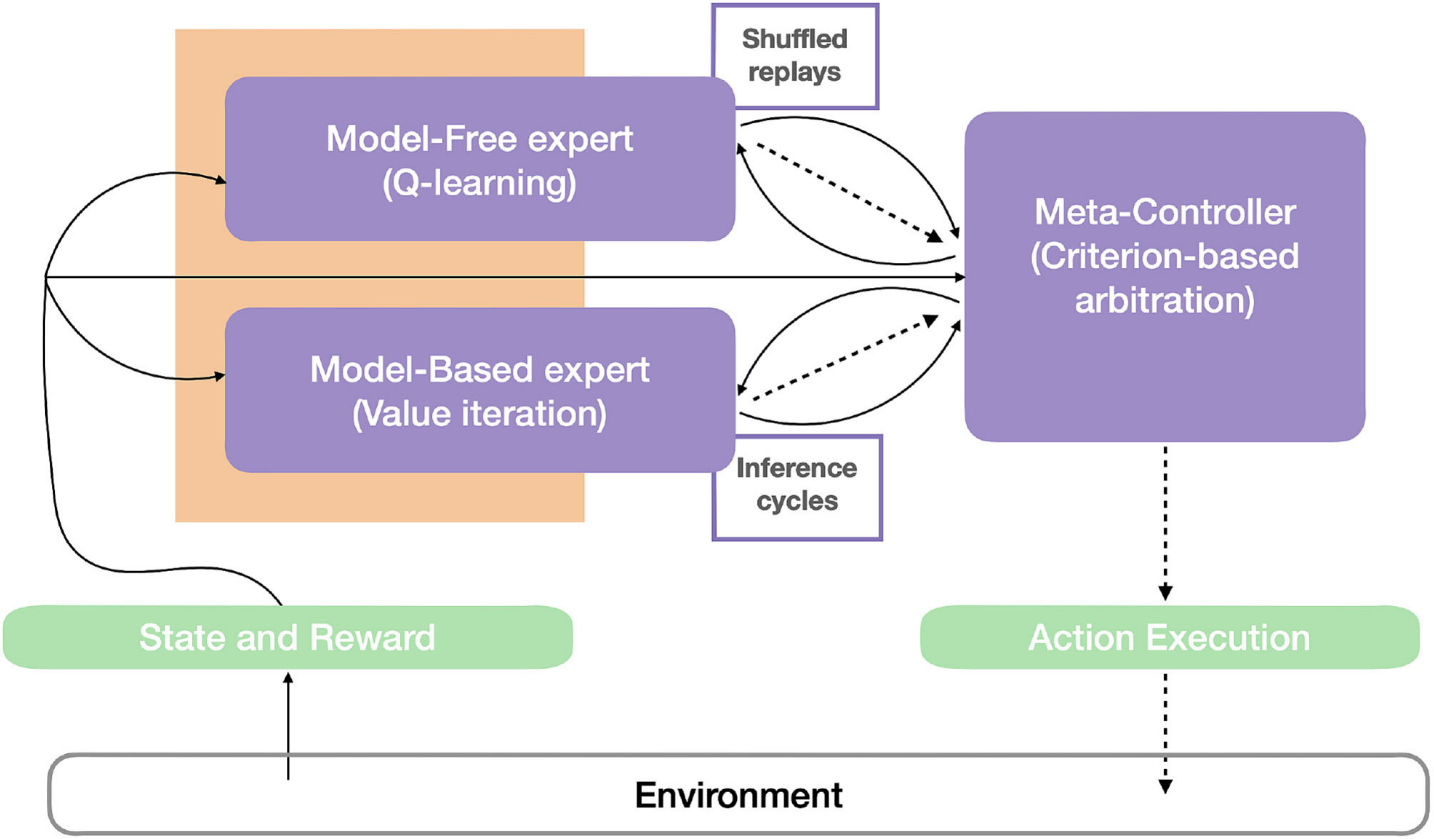
Robot control architecture. The agent-environment interaction can be described by (1) the state and the reward, as perceptual information (continuous arrows) from the environment, and (2) by the action (dashed lines) that the agent operates in the environment. The perceptual information is used by the model-free, the model-based expert, and the meta-controller (in purple). Based on this information and memory of their previous performances, the meta-controller estimates the entropy and computational cost of the experts, consistently with the criterion in Equation 13, and thus choose the expert that will be allowed to infer the probability distribution of the next agent's actions. This distribution, and the times consumed to compute it (dashed arrows), are then sent to the meta-controller. Different from Dromnelle et al. (2020b), both experts here have a 'replay' (reactivation) budget (limited or until convergence) that will affect both their performance and computation time and thus impact the meta-controller's arbitration. Here, shuffled memory reactivations (MemR) are integrated with the Q-learning algorithm of the MF expert, while simulation reactivations (SimR) constitute the offline MB inference iterations in the value iteration algorithm of the MB expert.

The robot control architecture proposed in Dromnelle et al. (2020b) and also successfully applied to a simulated human-robot interaction task in Dromnelle et al. (2020a) takes inspiration from the mammalian brain's ability to coordinate multiple neural learning systems. Such ability is indeed considered to be key to making animals able to show flexible behavior in a variety of situations, to adapt to changes in the environment, while at the same time minimizing computational cost and physical energy (Renaudo et al., 2014). The proposed architecture in Figure 7 is composed of a decision layer where a MF expert and a MB expert compete to determine the next action of the system. Both experts pass through three different phases: learning, inference, and decision. A meta-controller (MC) determines which proposed decision will be executed, following an arbitration criterion that we describe below.

#### 4.1.1. Model-Based Expert

The model-based algorithm is implemented to learn a transition model *T* and a reward model *R* of the specific task. Thanks to these two learned models, it can predict the consequences of a given action several steps ahead and can adapt faster to non-stationary environments. Yet these computations are very costly (i.e., 1,000 times higher than the computations of the MF expert in Dromnelle et al., 2020b).

During the *learning process*, the transition model and the reward model are updated at each timestep after observing the departure state *s* of the robot, the action *a* that it has performed, the arrival state *s*′, and the scalar reward *r* that this transition may have yielded. The transition model is updated by estimating *T*(*s, a, s*′), the probability of arriving in *s*′ from (*s, a*), considering the past *T*_*tw*_ actions (Table 3). This probability is computed as already shown in Equation 3. Besides, the reward model *R*(*s, a, s*′) is updated by considering the most recent reward *r*_*t*_ associated to the transition (*s, a, s*′), multiplied by the probability of the transition itself in Equation 3.

**Table 3:** Parameters used to generate the results in this section.

	Model-based	Model-free	Meta-controller
α	-	0.6	-
γ	0.95	0.9	-
β	50	50	50
ϵ	0.01	0.1	-
*RB*	-	100	-
*R*_*tw*_	-	100	-
*T*_*tw*_	30	-	-

*They are taken from Dromnelle et al. (2020b) as a starting point for this work. α is the learning rate, γ is the discount factor, and β is the exploration/exploitation trade-off parameter. For the MF expert, the converge threshold ϵ and replay constant RB have been introduced to design the convergence criterion, while ϵ for the MB expert is the same as in Dromnelle et al. (2020b). R_tw_ is the number of the last (s, a, s′, r) tuples that the MF expert can replay. T_tw_ is the number of the last (s, a, s′, r) tuples considered to built the transition model T for the MB expert*.

The *inference process* estimates the action-value function *via* the value iteration algorithm (Sutton and Barto, 1998), and it operates as an offline planning phase that is continuously called every decision step, just before a decision is made by the agent about which action to perform. The maximal duration of this planning process can be determined either by setting a finite budget for the number of transitions over which the agent will evaluate its decision or by employing a convergence criterion based on the sum of the absolute action-value function estimation errors. More precisely, the planning terminates at iteration *c* if


(7) $$\begin{array}{r} \underset{s,a}{\sum }|\delta_{s,a}^{c}|<\epsilon_{MB}\text{ where} \end{array}$$

(8) $$\begin{array}{r} \delta_{s,a}^{c}=\underset{s^{\prime }}{\sum }p\left(s^{\prime }|s,a\right)\left[R_{s,a}^{c}+\gamma V{\left(s^{\prime }\right)}^{c}\right]-Q{\left(s,a\right)}^{c} \end{array}$$

Here, $R_{s,a}^{c}$ is the reward function of performing action *a* from state *s* at the offline reactivation *c* and *V*(*s*′) is the value function of the arriving state *s*′ at reactivation *c*, from state *s* and action *a*. γ is the discount factor (Table 3).

Finally, the *decision process* chooses the next action to be performed by the robot by converting the action-value function into a probability distribution using a softmax function (see Equation 2), with an exploration/exploitation trade-off parameter β given in Table 3.

#### 4.1.2. Model-Free Expert

The model-free algorithm does not learn any transition or reward model of the task, in contrast to the MB expert. Rather, it locally updates the current action-value function *Q*(*s, a*) at each timestep. This property of the MF expert makes it save computational cost, compared to the MB expert, at the expense of slow adaptability to task changes, given the expert's lack of topological knowledge of the environment.

The *inference process* simply consists of reading from the Q-table the line corresponding to *s* which is then used by the *decision process*. The latter chooses the next action from the Q-values, also converted to a probability distribution with a β trade-off parameter in Table 3.

For the MF-RL expert, the *learning process* is defined as a tabular Q-learning algorithm in which the action-value function *Q*(*s, a*) is updated according to Equation 1. Following the online learning phase, *shuffled replay* is performed, using the (*s, a, s*′, *r*) tuples experienced by the agent in a given time-window of past transitions *R*_*tw*_ (Table 3). As for the MB expert, these offline updates stop when either the maximal predefined budget is exhausted or when the Q-values have converged. Since the MF expert does not know the transition probabilities of the task, a convergence test is computed for every offline learning iteration *c* as in Equation 9, where $act_{s,a}^{c}=\tau \cdot act_{s,a}^{c-1}$, with $act_{s,a}^{{\tilde{c}}_{s,a}}=RB$ during the first time ${\tilde{c}}_{s,a}$ when that specific transition is selected for replay and with $act_{s,a}^{0}=0$. *act* is an activation function defined for each couple (*s, a*), and it is 0 if (*s, a*) has not been replayed before or otherwise it decays from *RB* (Table 3) along the replay iterations *c* with a time constant τ (Equation 11).


(9) $$\begin{array}{r} \underset{s,a}{\sum }\delta_{s,a}^{c}act_{s,a}^{c}<\epsilon_{MF}\text{ where} \end{array}$$

(10) $$\begin{array}{r} \delta_{s,a}^{c}=|Q{\left(s,a\right)}^{c}-Q{\left(s,a\right)}^{c-1}| \end{array}$$

The principle behind the design of this convergence criterion is that the importance of each δ_*s,a*_ (Equation 10) starts as *RB* and decreases over the offline learning iterations *c*, following the decay constant τ (Equation 11). This strategy does not constrain the number of needed replay iterations, because the agent would still perform replays due to high $\underset{s,a}{\sum }\delta_{s,a}^{c}act_{s,a}^{c}$. Nevertheless, this value will slowly decrease the need for more replay iterations along with the offline learning phase. *RB* is a value representing one of the possible replay budgets needed to obtain performances that are comparable to the maximum amount of reward that the expert can collect, thus not inhibiting the offline learning phase when needed. Finally, the convergence threshold ϵ_*MF*_ is an order of magnitude larger than ϵ_*MB*_ (Table 3 which is the same used in Dromnelle et al., 2020b). The MF expert does not know the probabilities contained in the transitions model in Equation 3 and for this reason, its convergence criterion is based on the actual update of the action-value function *Q*(*s, a*). This means that, in the MF case, the $\delta_{s,a}^{c}$ are not multiplied by any probability, derived from the world model, and thus their values will usually be an order of magnitude larger than the $\delta_{s,a}^{c}$ of the MB case, multiplied instead by the probability of a given (*s, a, s*′, *r*) tuple.


(11) $$\begin{array}{r} \tau =\sqrt[{RB}]{\frac{\epsilon_{MF}}{RB}} \end{array}$$

#### 4.1.3. Meta-Controller

The meta-controller selects, which expert will take the control of the next action, by following a specific criterion that is a trade-off between the learning performances and the computational cost of the inference process of the two agents and it is called *Entropy and Cost (EC)* (Dromnelle et al., 2020b).

On the one hand, the quality of learning is computed by Equation 12 where *f*(*P*(*a*|*s, E, t*) is a low-pass filtered action probability distribution with a time constant τ = 0.67, previously used as an indicator of the learning quality in humans (Viejo et al., 2015).


(12) $$\begin{array}{r} H_{exp}\left(s,E,t\right)=-\overset{\left|A\right|}{\underset{a=0}{\sum }}f\left(P\left(a|s,E,t\right)\right)\cdot \log_{2}\left(f\left(P\left(a|s,E,t\right)\right)\right) \end{array}$$

On the other hand, the cost of the process *C*(*s, E, t*) is the computation time needed to perform the inference phase for the expert *E*, at time *t*, and it is also filtered as the action probability distribution above.

Eventually, the MC chooses which expert will take control of the next decision by following the equation below (Dromnelle et al., 2020b):


(13) $$\begin{array}{r} EX\left(s,E,t\right)=-\left(H_{exp}\left(s,E,t\right)+\kappa C\left(s,E,t\right)\right) \end{array}$$

*EX*(*s, E, t*) is the expertise value of the expert *E*, which is then converted into a distribution of probabilities using a softmax function. κ weights the impact of time in the criterion by assigning greater importance to the computation time when the entropy component *H*_*exp*_(*s, E, t*) of the MF experts is low.

After applying Equation 13, the MC draws the winning expert from the softmax of the distribution of their expertise *EX*(*s, E, t*) (with a trade-off coefficient β shown in Table 3) and inhibits the inference process of the expert that is not selected.

#### 4.1.4. The Experimental Set-Up and Implementation

**Figure 8:**
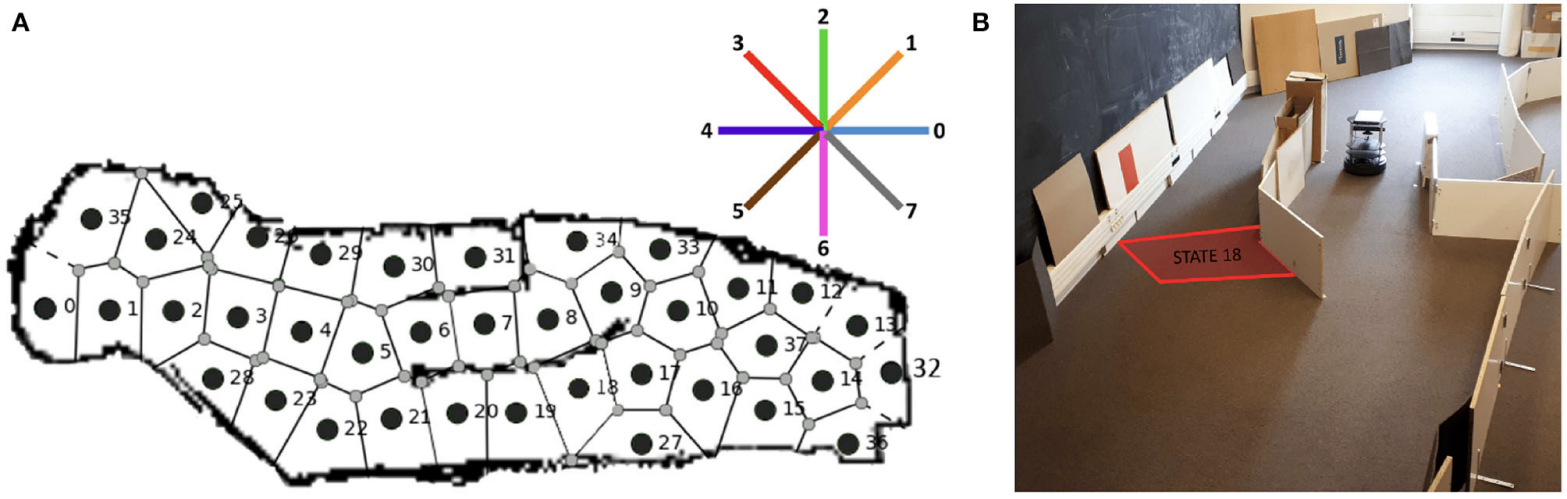
Description of the experimental set-up. **(A)** Map of the discrete states of the maze. The eight-pointed star indicates the cardinal directions in which the robot can move. These directions are the same used for the experiment in Section 3 **(B)** Photo of the real Turtlebot approaching the initial rewarding state 18, highlighted in the figure. Adapted from Dromnelle et al. (2020b).

This new hybrid MB-MF RL architecture with replay is tested in a dynamic navigation task where the robot has to learn how to reach a unitary rewarding state. The task remains stationary during the first 1,600 over 4,000 iterations and then the reward is moved to another state (from state 18 to state 34, Figure 8). In this experiment, an extra element of non-stationarity is represented by the starting state of the robot being uniformly selected with the same probability between state 0 and state 32 at the beginning of each trial (**Figure 10**). Different from Dromnelle et al. (2020b), experiments where the reward is fixed or where a new obstacle is introduced have not been performed for this work.

First, the real Turtlebot autonomously navigates within the environment using a SLAM Gmapping algorithm (Figure 8) and creates a discrete map of the maze (38 Markovian states are identified and shown in Figure 8A). This autonomous state decomposition process is identical to the one used in the previous experiment described in Section 3.1.2. The robot-environment ratio is very similar to the one of the previous experiment in Section 3.1.2: the state radius is 35 cm, in this case, and the robot size is 35,4 x 35,4 x 42 (L x W x H, cm).

Then, during a second free exploration phase, the robot learns the transition model of the environment, that is, the probability that the robot starts its move in one state *s* performs an action *a* and arrives in another state *s*′. This second phase of the creation of the transition model is also conducted as in Section 3, but with the real robot in this case.

After these exploration phases, the subsequent experiments involving a reward were performed in simulation to test the impact of different parameters of the algorithm and study the effect of replay on total performance and computation cost. During these simulations, the agent experienced the MDP based on the transition map that was empirically acquired with the real robot (as was done in Dromnelle et al., 2020b).

**Figure 10B** shows the maximum level of uncertainty for each of the 38 states of the environment. This uncertainty is computed in the same way as for the other experiment in Equation 5, and the transitions map is used to guide the robotic exploration in the simulation environment.

The action space is also discrete and consists of 8 possible cardinal directions equally distributed around the agent. Given the discrete and probabilistic nature of the state and action spaces, the transition model *T*(*s, a, s*′) (Equation 3) and the reward model *R*(*s, a*) of the MB expert are probability distributions.

### 4.2. Results

To evaluate the contribution of combining MB and MF replay in terms of performance and computational cost, we tested several algorithms. First, we are interested in simulating the two baseline cases, pure MF and pure MB algorithms, and how they perform with the respective MemR and SimR and limited budgets. Finally, we want to test the combination of the two strategies by using the criterion proposed in Dromnelle et al. (2020b), with either an infinite or a limited reactivations budget. Here are the relevant combinations of the same controller that we tested in this task:

- MF only agent, no replay
- MF only agent with MF replay (infinite replay budget)
- MF only agent with MF replay (budget: 200 replay iterations)
- MB only agent with MB replay (infinite inference budget)
- MB only agent with MB replay (budget: 200 inference iterations)
- MB+MF agent with MB replay (infinite inference budget)
- MB+MF agent with MB budget (budget: 200 inference iterations)
- MB+MF agent with MF replay (budget: 100 replay iterations) and MB replay (budget: 100 inference iterations) (a fair comparison with the previous cases because here the reactivation buffer is split in a maximum of 100 iterations per expert)

All the MB+MF agents use the EC coordination criterion described in Section 4.1.3. This criterion was taken from Dromnelle et al. (2020b) which showed that it allows for advantageous coordination between MB and MF experts and significantly reduces the computational cost of the inference phase, without relevantly impacting the amount of gained reward. Table 3 shows the values of the parameters that we used for these experiments.

**Figure 9:**
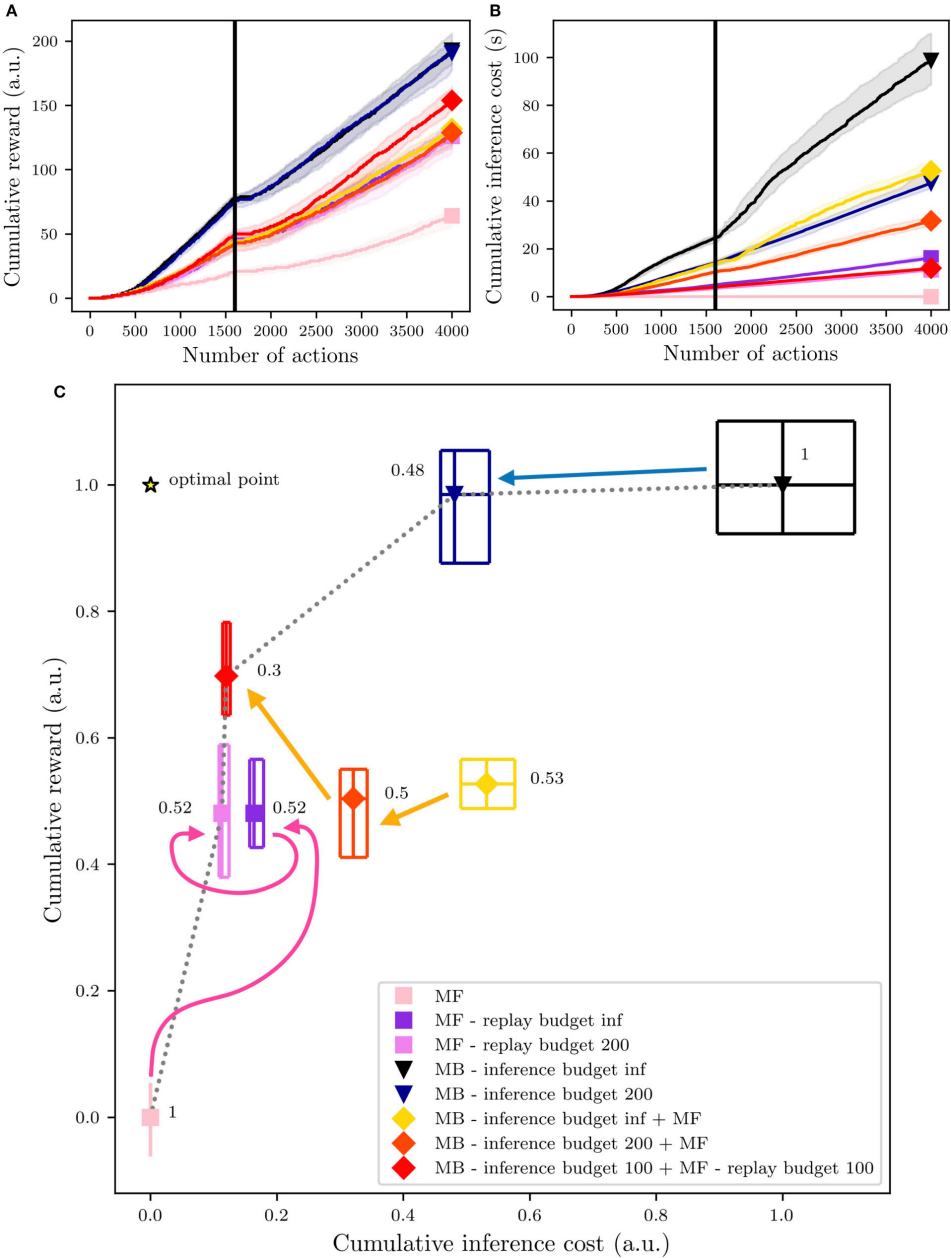
Overall performances of the different agents during their first 4,000 actions in the environment. The vertical black line highlights the trial when the reward switch (1,600). **(A)** The dynamics of the reward's accumulation. **(B)** The dynamics of the computational cost's accumulation. **(C)** An overview of the algorithms' position within a normalized reward × cost space. The central polygons represent the median of the performance over 50 simulated experiments. Cumulative rewards and costs have been normalized considering that the MF medians of the cumulative rewards and costs correspond to 0 and that the MB medians of cumulative rewards and cost correspond to 1.

The speed of learning of all the above-listed agents was impacted when the reward's position changed at iteration #1600 (Figure 9A). It is interesting to notice that the *MB - inference budget 100 + MF - replay budget 100* agent, which exploited the EC criterion with a limited budget for the two experts, shows a faster increase in the cumulative reward compared to all the other agents, from around actions #2500. As observed in the previous experiment (Section 3), replay contributes to increasing the speed of learning and by combining the action of both MF and MB replay, it is possible to better account for both adaptability and generalization, drastically leading to a steeper accumulated reward over time slope of the proposed strategy, without having the same growth on the computational cost side (Figures 9A,B). Concerning the cumulative costs, Figure 9B shows that it rapidly increases for the *MB - inference budget inf* agent when the environment changed, and eventually, by action #4000, its cumulative cost has doubled the ones of the other agents.

Thus, considering the final overview of the performances and computational costs in Figure 9C, deeper analyses and comparisons of the tested algorithms can be presented. The results are represented in terms of first, median, and third percentiles over 50 experiments. The cumulative reward is the amount of reward each agent has accumulated over the entire experiment, which is composed of 4,000 iterations of the learning, inference, and decision processes together (Section 4.1). The cumulative inference cost represents the time (in seconds) needed to perform the inference phase.

As expected, reward-wise, the best performing agent is the pure MB, with an infinite inference budget (black triangle, on the top-right, in Figure 9C). However, this agent is also the most costly in terms of computation during the inference phase. This issue can be partially fixed by reducing the MB replay budget to 200 iterations (blue triangle, in Figure 9C). In this case, the inference phase will be stopped either if the action values have converged or if the number of inference iterations has reached the maximal budget (in this case 200).

On the opposite side of the figure, the pure MF agent (pink square, on the bottom-left, in Figure 9C) shows the minimum cost of the entire set of experiments, but also the lowest cumulative reward. Adding replay to the MF expert, with an infinite replay budget (dark violet square in Figure 9C) or a 200-iteration budget (light violet square in Figure 9C) doubles the reward accumulation performance, with a limited increase in the computational costs (compared to the MB costs), in particular when adding the budget of 200 iterations.

From the results in Figure 9C, we can deduce that for both the MF and the MB experts, most of the time, the number of needed reactivations is in the same order of magnitude as the proposed finite budget of 200 (since the cumulative costs are comparable). As already shown in Dromnelle et al. (2020b), with an MB expert with an infinite inference budget, the coordination of MB and MF experts *via* the EC criterion produces agents which are halfway between MB-only and MF-only experts, regarding performances and costs (yellow diamond in Figure 9C). Nevertheless, when limiting the MB inference budget to 100 and adding the contribution of 100 replay iterations for the MF expert (red diamond in Figure 9), the cumulative reward increases, and the inference cost diminishes, moving the performance of the agent closer to the optimal point (star in Figure 9). Moreover, the arrows highlight the progressions of the *MF-only* (pink), the *MB-only* (blue), and the *MB+MF* (orange) agents. Looking in more detail, the performance of the MF-only agents is improved by adding a budget of 200 MF replays and on the other hand, the performance of the MB-only agents is slightly decreased by limiting the inference budget to 200 iterations, but the cumulative computational cost is significantly decreased. Starting from the performance obtained in Dromnelle et al. (2020b), in yellow in the figure, we obtain similar performances but decrease the computational cost when we limited the inference budget to 200 inference iterations for the MB expert, producing agents which are halfway between MB-only and MF-only experts. After this analysis, we have tested the combination of the two best strategies tried so far: the MB expert with a limited inference budget and the MF one with a limited replay budget and we have combined them through the EC criterion (Equation 13). In this case, to have the same total reactivations budget as the other tested algorithm, we have shared the initial 200 reactivations budget to 100 SimR for the MB expert and 100 MemR for the MF one. With this combined replay effort, the overall performance reached an optimal compromise between performance and cost since the inference cost is substantially decreased while the cumulative reward was significantly raised, compared to the results obtained by Dromnelle et al. (2020b).

Given that the aim of each agent and its EC meta-controller is composed of two objectives: (1) maximizing the cumulative reward and (2) minimizing the cumulative inference cost, we compute the pareto front (black dotted line in Figure 9C), which represents the solutions that approximate the set of all optimal trade-offs of the two given objectives. As expected, the pure MB and MF experts are pareto optimal solutions, very specialized in one of the two objectives, while by reducing and splitting their budgets we can have agents that interestingly converge closer to the *OptimalPoint* (star in Figure 9C). To rank all the agents *ag*, the Chebyshev distance (Cantrell, 2000) from their median performance to the *OptimalPoint* is computed as shown in the following equation:


(14) $$\begin{array}{r} Chebyschev\;distance\;\left(ag\right)=\underset{obj}{\max}|OptimalPoint_{obj}-median\left(ag_{obj}\right)| \end{array}$$

where *obj* are the 2 normalized objectives of the solutions space (cumulative inference costs and cumulative reward). The computed Chebyshev distances are shown in Figure 9C, on the side of each algorithm point, and show a clear picture concerning the proposed solutions; the agent sharing the reactivations budget between the MB and MF is the closest to the optimal point, followed by the MB expert with limited SimR budget. MF with MemR and MB + MF without MemR have very similar distances to the optimal points, meaning that the contribution of the MB expert is key to adapting to a dynamical environment, but the cost of this computation can largely decrease just when it cooperates with an MF agent with replay, that can learn faster also from the Q-values update of the MB expert.

These results open new possibilities for the design of RL control architectures in robotics. When dealing with probabilistic environments, MF replay might focus mainly on rare and not relevant transitions, leading to interesting exploration and computational economy, but misguiding the memory consolidation of relevant experience, when changes happen in the task (as also seen in Section 3). When the transitions model is stochastic, the combination of the computationally competitive MF replay with the general knowledge of the environment, acquired by MB replay, can bring artificial agents and robots to better deal with a non-stationary RL task.

## 5. Discussion

In this paper, our research question was whether RL strategies using neuro-inspired replay methods, based on neuroscience knowledge about the hippocampal replay, could improve the speed and the adaptability of robotic agents engaged in spatial navigation tasks. MF, MB, and no replay RL techniques were compared in three simulated robotic experiments of increasing complexity and realism. Our results showed that in all levels of abstraction, the neurorobots learned the spatial task faster when the replay was involved in the process, and more efficiently when a MB method replay method was used. Conversely, we show how a synergy between MB and MF replay methods can be more effective in a more realistic and stochastic experimental setup.

The application of RL techniques to robotics requires coping with some specificities of operating in the real world (Kober et al., 2013). First, making actual movements in the real world takes time, wares out the robotic platform, and also has the potential of damaging it. Acquiring new data requires the robot to move, and thus to incur those costs. Online learning processes therefore have to be as much parsimonious on data use as possible. Second, making decisions also takes time, especially when using limited embedded computation systems, while operating in a dynamic world may require the ability to react extremely rapidly to avoid damage. Learning systems should thus be as computationally cheap as possible. Finally, moving and computing both consume the robot's energy, which is always available in limited amounts. This highlights the importance of developing robotic controllers that can (1) maximize their learning capabilities over experience and energy scarcity and (2) reduce the complexity of their algorithm to meet the computational limitations of embedded platforms.

All along with this paper, we have presented simulated experiments (sometimes based on data like transition maps first generated with a real robot) to investigate the possible advantages of equipping neurorobots with offline learning mechanisms inspired by hippocampal place cells' reactivations. These advantages are, first, to extract as much information as possible from the already gathered data, and, by mixing the multiple types of learning processes with the multiple types of reactivations, to limit deliberation time, and to limit the aforementioned costs intrinsic to robotics. Starting with simpler and deterministic environments, as the double T-maze experiment presented in Section 2, this research illustrates that as the complexity of the state-action spaces increases, MB *SimR* become more strategic for the learning capabilities of the agent (Section 3). In Section 4, the combination of MF *MemR* and MB *SimR* is presented as an interesting proposal to merge the benefits of both techniques: prioritizing the MB expert when the task requires more inference and generalization effectiveness to be solved (for example facing non-stationarity), while on the contrary giving priority to the MF expert when an effective solution can be found relying only on recent experience.

**Figure 10:**
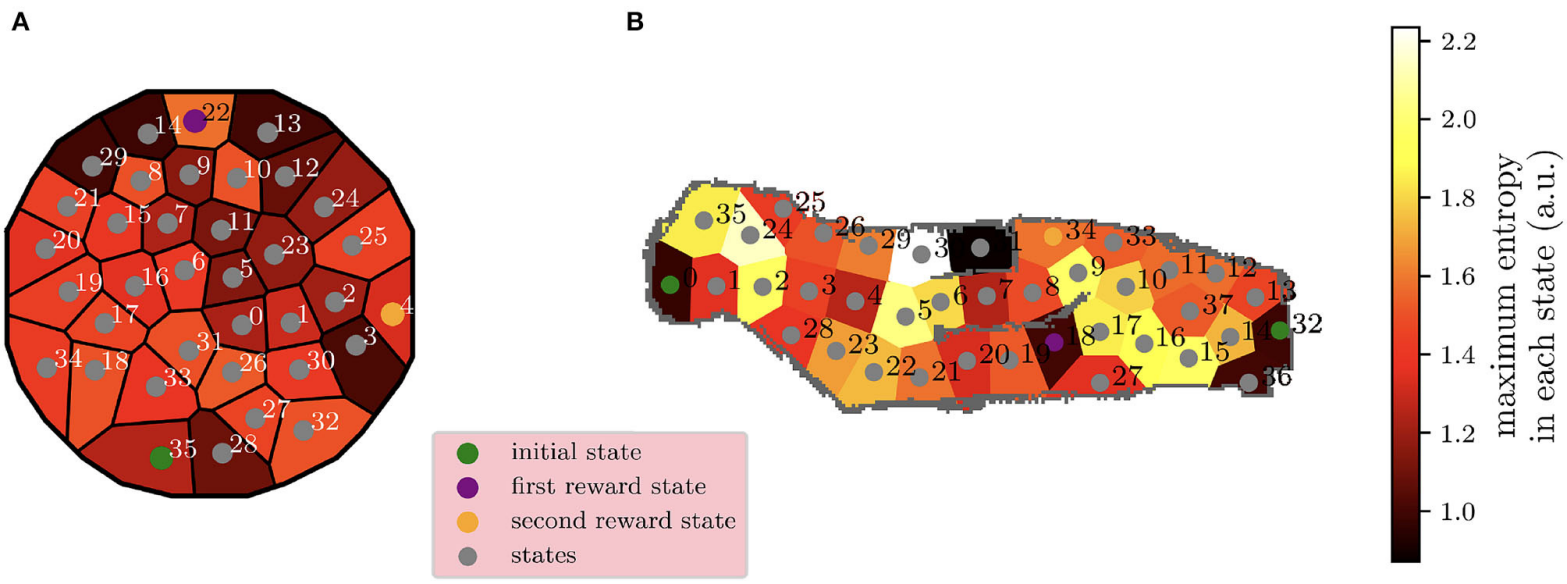
Representation of the navigation environments for the previous experiments, Section 3 and Section 4, organized in, respectively, 36 and 38 discrete Markovian states decomposed from the data acquired during the autonomous navigation of the robot, when no reward was present in the mazes. The initial and reward states for the tasks are also highlighted in the figure. In these heatmaps, the lighter the color of the state, the greater the maximal entropy of that specific state, according to Equation 5. The represented scale of entropy values (0.87–2.23 a.u.) has been selected to cover the whole range of the computed entropies. Moreover, in both environments, the robots have navigated for 5357 actions. **(A)** In the case of the circular maze (Section 3), the navigation and the transition model are acquired after simulated navigation on ROS Gazebo. **(B)** In the second experiment (Section 4), the navigation and the transition model are instead computed after the real robot navigation, which generated a wider range of maximal entropy values, sometimes also very low due to the presence of walls that categorically constrained certain states of the environment.

When simulations increase in complexity, thus getting closer to a real robotic experiment, the challenges regarding the internal representation of the world (in particular the state-action space and the reward) increase. As presented in Figure 10, where the environments of the two last experiments (presented in Sections 3, 4 respectively) are displayed in terms of maximum entropy per state, it is visible that the transition probability matrix created by the navigation of the real robot (Figure 10B) results in a representation of the environment which is less homogeneous and more uncertain than the one learned with the simulated robot (Figure 10A). Often, in mobile robotics, localization may depend on a few sensory information, as in the case of the mobile robots used in our experiments. Such limited information is however fundamental for the acquisition of a solid representation of the environment. For these reasons, the entropy maps in Figure 10 reflect the nature of the two mazes: the uncertainty is more homogeneous in the circular maze (Figure 10A) since the environment is an open space which gives the agent an even chance to end up visiting the neighboring states. In contrast, the second environment (Figure 10B) is longer in one dimension and presents inner walls that result in a fuzzier level of uncertainty on the transitions model of the environment.

Future works in this research direction would include the comparison with the RL algorithms performing forward replays, which are of crucial importance in standard rodents navigation tasks, such as the multiple T-maze (Johnson and Redish, 2007). These forward-shifted spatial representations have been demonstrated to happen largely at decision points to predict the consequences of the next actions. Their effect has already been successfully modeled in neurorobotics by Maffei et al. (2015), where they implemented the extractions of relevant policies by consulting memory. On the other hand, van Seijen and Sutton (2015) argued that it is mathematically equivalent to update Q-values in a MF way combined with replay and to update Q-values in a MB way, given that the elements in the memory buffer, used for replay, are the same than those used to build the model. Moreover, RL-based replay strategies can also generate forward replay events (Khamassi and Girard, 2020) and enable RL-based models to still account for neurobiological data (Cazé et al., 2018; Mattar and Daw, 2018).

In summary, this work presented new and crucial results concerning the advantages and the limitations of different RL-based replay techniques for robotics, gradually testing them in more and more complex and realistic circumstances. Additionally, this research paves the way for new studies on the role of replays in neurorobotics, in particular, in spatial navigation tasks where generalization effectiveness and time efficiency are key.

Finally, the addition of RL techniques, inspired by hippocampal replays, shows an improvement in the performance of the presented navigation task, in particular, concerning the exploitation of the past experience, knowledge propagation, and as a consequence, the speed of learning. MB *SimR* significantly contributed in the case of non-stationarity, but a fruitful coordination with MF *MemR* became crucial in terms of computational cost reduction. All these insights, found in robotic experiments, implemented with different levels of abstraction, can encourage new neuroscientific experimental protocols and shed light on a better understanding of the phenomenon of hippocampal replay.

## Data Availability Statement

The original contributions presented in the study are included in the article/supplementary material, further inquiries can be directed to the corresponding author.

## Author Contributions

BG and MK: conceptualization, project administration, and funding acquisition. EM, JB, JM, RD, BG, and MK: methodology. EM, JB, JM, RD, JC, EP, and MK: software. EM and MK: validation and writing—original draft. EM and JB: formal analysis. EM, JB, JM, BG, and MK: writing—review and editing. EM, JB, JM, EP, and MK: visualization. EM, BG, and MK: supervision. All authors contributed to the article and approved the submitted version.

## Funding

The authors acknowledge the funding of the CNRS 80|PRIME program, RHiPAR project, the French National Research Agency ANR, and the Délégation Générale de l'Armement (RD).

## Conflict of Interest

The authors declare that the research was conducted in the absence of any commercial or financial relationships that could be construed as a potential conflict of interest.

## Publisher's Note

All claims expressed in this article are solely those of the authors and do not necessarily represent those of their affiliated organizations, or those of the publisher, the editors and the reviewers. Any product that may be evaluated in this article, or claim that may be made by its manufacturer, is not guaranteed or endorsed by the publisher.

## Footnotes

^1^The updated code for these simulations is available at https://github.com/MehdiKhamassi/RLwithReplay.

^2^The code for these simulations is available at https://github.com/esther-poniatowski/Massi2022.

^3^The code for these simulations is available at https://github.com/elimas9/combining_MB_MF_replay.
